# Household Water Treatment and Cholera Control

**DOI:** 10.1093/infdis/jiy488

**Published:** 2018-09-11

**Authors:** Daniele Lantagne, Travis Yates

**Affiliations:** Department of Civil and Environmental Engineering, Tufts University, Medford, Massachusetts

**Keywords:** cholera, chlorination, filtration, household water treatment, point-of-use water treatment

## Abstract

Water, sanitation, and hygiene are one part of a cholera control strategy. Household water treatment (HWT) in particular has been shown to improve the microbiological quality of stored water and reduce the disease burden. We conducted a systematic review of published and gray literature to determine the outcomes and impacts of HWT in preventing cholera specifically. Fourteen manuscripts with 18 evaluations of HWT interventions in cholera were identified. Overall, a moderate quality of evidence suggests that HWT interventions reduce the burden of disease in cholera outbreaks and the risk of disease transmission. Appropriate training for users and community health worker follow-up are necessary for use. Barriers to uptake include taste and odor concerns, and facilitators include prior exposure, ease of use, and links to preexisting development programming. Further research on local barriers and facilitators, HWT filters, scaling up existing development programs, program sustainability, integrating HWT and oral cholera vaccine, and monitoring in low-access emergencies is recommended.

In the late 19th and early 20th centuries, epidemic cholera was virtually eliminated in industrialized countries by the introduction of municipal water supply with treatment and sanitation infrastructure [1]. A century later, in 2015, 844 million people lack access to a basic water service, and 25% of the global population drinks microbiologically contaminated water [2]. Within this inadequate water and sanitation context, cholera transmission continues.

In 2016, 38 countries—many of which are struggling with poverty, rapid population growth, and instability—reported cholera transmission [3]. Until there is universal access to reliable piped-on-premises water, reducing the remaining cholera burden requires a multipronged strategy. Household-level water, sanitation, and hygiene (WASH) interventions are one part of that strategy [4]; in particular, WASH interventions such as household water treatment (HWT) can provide heath gains associated with safer drinking water until more permanent supply or treatment solutions are available.

HWT methods can be broadly grouped into 5 technologies: (1) coagulation, flocculation, and sedimentation; (2) filtration; (3) chemical disinfection (eg, chlorination); (4) disinfection by heat, ultraviolet radiation, or solar radiation; and (5) combined methods. A growing body of evidence demonstrates that HWT use improves the microbiological quality of household water and reduces the burden of diarrheal disease [5].

In 2012, a literature review and survey of implementers on HWT in emergencies was conducted [6]. HWT was found to be effective in small-scale, nonacute, high diarrheal disease–risk emergencies when training and materials were provided to recipients, adequate product stocks were maintained, and chlorine dosage was appropriate. Of critical note, there was little documented effectiveness in acute emergencies or during large-scale distributions without training.

In 2015, a systematic review on WASH evidence in cholera was completed [7]. This review included peer-reviewed manuscripts with a cholera health outcome or data on the function or use of the intervention. Eight studies included an HWT intervention, including filtration, solar disinfection (SODIS), and chlorination products. While HWT was the most reported intervention in the review, 3 of the 6 HWT studies reported inconsistent product use. It was recommended that HWT be accompanied by health education so sustainable behavior change could be achieved.

In the current manuscript, we present results from a specific broad review of the evidence for HWT in cholera outbreaks, using both published and gray literature, and investigating 3 research questions:

1. What are the health impacts of HWT products in cholera outbreaks?
2. What are important HWT program design and implementation characteristics in cholera outbreaks?
3. What are the population-related barriers and facilitators that affect HWT interventions in cholera outbreaks?

## METHODS

We previously conducted a systematic review of published and gray literature on the evidence of WASH in outbreaks, including development of theory of change models, search strategy, inclusion criteria, selection and processing strategy, quality of evidence appraisal, and analysis plan. Please note that the full systematic review protocol was peer-reviewed and made publicly available before conducting the review [8], and full review results have been published previously [9]. For the current manuscript, evaluations specifically related to HWT and cholera were extracted from [8] and [9]. Each step of the review process is summarized below.

### Theory of Change Model Development

**Figure 1.**
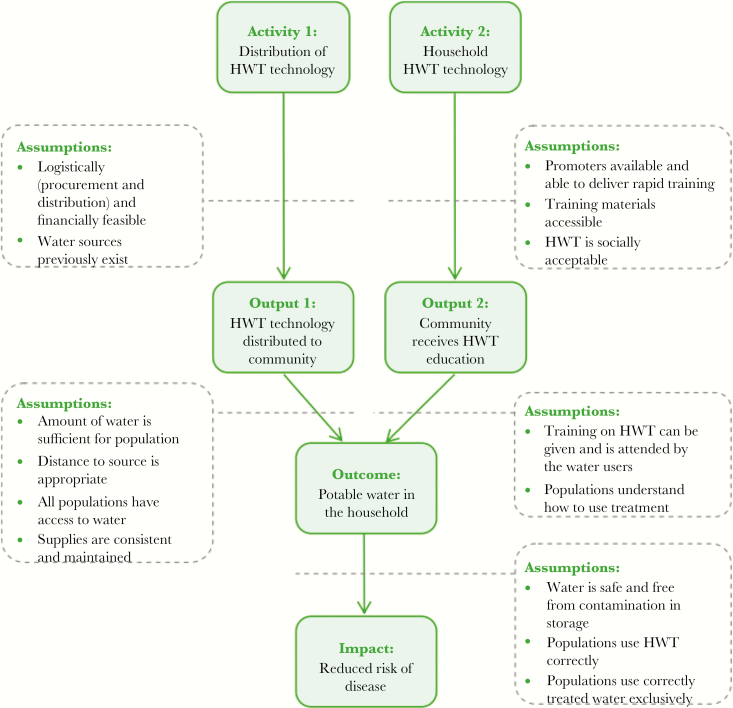
Theory of change for household water treatment (HWT) in cholera.

A theory of change model for household water treatment was developed to describe the theoretical route from intervention activities to outputs, outcomes, and impacts, while also identifying influencing factors and assumptions [8] (Figure 1).

### Search Strategy

Using the theory of change, search strings were developed to identify published and unpublished gray literature. The search strings were used in 9 peer-reviewed databases and 10 searches, in English (7), French (2), and English/Spanish (1) including Cochrane Library, Google Scholar, IDEAS (economic literature), Latin American and Caribbean Health Sciences Literature (LILACS), Ovid Medline (PubMed), Scopus, Web of Science, Academic Search Premier (English and French), and ArticleFirst. The full search was completed in September 2016. A specific search on HWT and cholera was rerun in April 2018. Additionally, 6 journals identified as most likely to have relevant research, reference lists of reviews identified, and responder websites were screened by hand. Last, solicitation for relevant documents was carried out via email and personal contacts.

### Inclusion Criteria

Inclusion criteria were established according to the PICOS (populations, interventions, comparisons, outcomes, and study types) framework [8].

#### Populations

All age, sex, and socioeconomic populations in low- and middle-income countries affected by an outbreak of cholera were eligible for inclusion. An outbreak was defined in accordance with World Health Organization definitions [10].

#### Interventions

A HWT intervention was eligible for review if it was field-based and began within 12 months of the start of the outbreak.

#### Comparisons

No specific comparisons were required.

#### Outcomes

Evaluations were included if at least one intermediate outcome (use of service or nonhealth outcomes) or final impact (disease reduction) were reported. Use of service included 3 specific indicators: reported use, confirmed use, and effective use. Reported use is when the beneficiary reported use without verification. Confirmed use is when an evaluator tests or observes use in some way (ie, testing free chlorine residual [FCR] in chlorine-based water treatment programs). Effective use is a measure of improving quality of contaminated water requiring confirmed use and microbiological testing. Disease reduction data were included if beneficiary morbidity and mortality impact were self-reported or clinically measured. Nonhealth outcomes of preferences from the population on use of interventions (eg, ease of use, taste of water), quality-of-life improvement (eg, feeling safer, time savings), and agency preferences for interventions were also included.

### Study Types

Experimental, quasi-experimental, nonexperimental, mixed-methods, and qualitative methodological study type designs were eligible for review.

### Selection and Processing

Identified studies were screened first by titles, then by abstracts and full texts. From abstract to final inclusion, studies were independently double screened by 2 authors. Discrepancies were discussed for final decision. Throughout the screening process, references were managed with EndNote X7 and Microsoft Excel 2010 software. Data collection was completed with a detailed coding sheet using Microsoft Excel 2010, and included author and publication details, type of intervention, context of the intervention, study design, study quality, effect estimation, outcomes and impacts, and barriers and facilitators to implementation.

### Quality of Evidence

Each included evaluation was assessed for the potential risk of bias. For quantitative studies, the bias assessment tool was based on the Cochrane Handbook “Risk of bias” tool and adjusted similarly to Baird et al [11, 12]. The risk of bias was assessed through 5 categories: selection and confounding; spillover effects and contamination; incomplete outcome; selective reporting; and other risks of bias. For qualitative studies, 4 appraisal categories were adapted from Spencer et al [13]: design, bias, data collection, and clarity of findings. Each category was scored as low risk, high risk, or unclear. The summary risk of bias for a study was based on the number of low-risk assessments across the categories.

To establish the summary quality of evidence from multiple studies of varying qualities and study designs, a protocol was developed based on the Grading of Recommendations Assessment, Development and Evaluation (GRADE) of evidence outlined in Cochrane Review Standards [11]. The summary of evidence was then described through 4 categories [14]: high evidence, further research is very unlikely to change confidence in the estimate of effect or accuracy; moderate evidence, further research is likely to have an important impact on confidence in the estimate of effect or accuracy and may change the estimate; low evidence, further research is very likely to change the estimate; and very low evidence, any estimate of effect or accuracy is very uncertain.

### Analysis Plan

The lack of experimental evaluations precluded statistical analysis; therefore, a narrative synthesis approach was used to summarize the information gathered. A summary of all included evaluations is first presented by country and type of literature. Then, a summary of evidence by HWT type is presented.

## RESULTS

Overall, >15000 documents were screened, and 14 manuscripts with 18 evaluations of HWT interventions in cholera were identified, including 8 manuscripts from peer-reviewed literature and 6 documents from gray literature (Supplementary Table 1). Nine countries were represented in this research, including Bangladesh, Democratic Republic of the Congo (DRC), Guinea-Bissau, Kenya, Liberia, Madagascar, Nepal, South Sudan, and Zimbabwe.

Overall, 11 evaluations of chlorine-based products, 3 flocculant/disinfectants, 2 filters, one SODIS, and one boiling were identified (Supplementary Table 1). HWT products were distributed as a sole intervention or included as one of several items in a hygiene kit, with and without associated hygiene promotion.

### Chlorine-Based HWT Products

Chlorine is often distributed in cholera response because it effectively inactivates the *Vibrio cholerae* bacteria, leaves residual protection, is low cost, and is easy to transport and use [15]. There are 2 chlorine-based HWT options used in cholera outbreaks: tablets and liquid. Users add one tablet or one measured capful to low-turbidity water, wait 30 minutes, and drink; higher-turbidity water can be treated by doubling this dose.

### Chlorine Tablets

**Figure 2.**
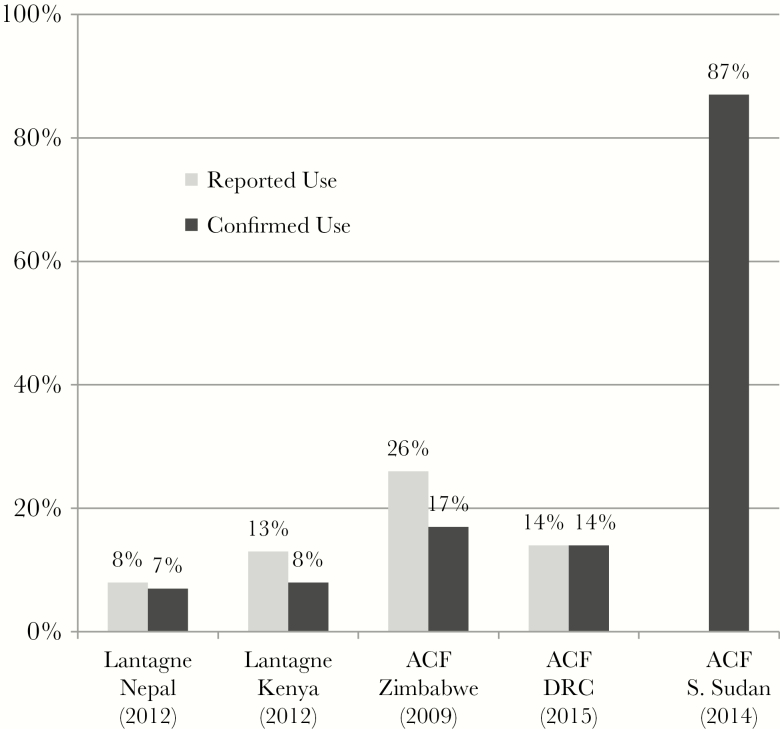
Chlorine tablet evaluations with reported and/or confirmed use. Abbreviations: ACF, Action Contre le Faim; DRC, Democratic Republic of the Congo.

Chlorine tablets were evaluated in 6 contexts in 5 studies, all where tablets were distribution for free in a hygiene kit [16–20]. Three evaluations in 2 studies were low risk of bias [16, 17], and 3 were high risk of bias [18–20]. Evaluations included both reported and confirmed use in 4 of 6 contexts [16, 17, 19], confirmed use in one context [18], and qualitative data only in one context [20] (Figure 2). Reported use ranged from 8% and 31% and confirmed use ranged from 7% and 87%. The outlier is a gray literature evaluation with a high risk of bias from a cholera response in South Sudan where hygiene promotion was conducted before HWT product distribution [18]. In this evaluation, 82% of the beneficiary population reported that drinking chlorinated water prevents cholera.

Knowing an HWT method before the outbreak was an indicator of use in Nepal [17]. The taste and smell of tablets was reported as a barrier to use in Zimbabwe and Nepal [17, 19, 20]. Taste/smell objections may have been because respondents did not have an appropriately sized water storage container for the tablet distributed, leading to confusion and overdosage.

### Liquid Chlorine

**Figure 3.**
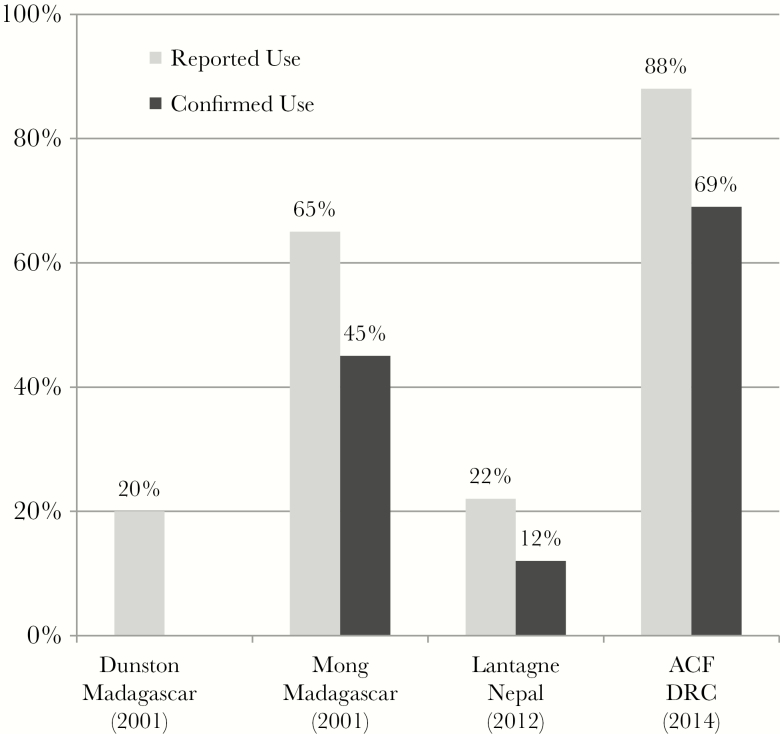
Liquid chlorine evaluations with reported and/or confirmed use. Abbreviations: ACF, Action Contre le Faim; DRC, Democratic Republic of the Congo.

Liquid chlorine interventions were evaluated in 5 contexts in 4 countries (Figure 3) [17, 21–24]. Three were high risk of bias [22–24], one was medium risk of bias [21], and one was low risk of bias [17]; one evaluation had only qualitative data [25]. Reported use in 4 studies ranged from 20% to 88%, and confirmed use in 3 studies ranged from 12% to 69% (Figure 3). It is noted that in the 2 studies with higher use rates [21, 23], the programs existed before the cholera outbreak and were scaled up as part of outbreak response activities. Overdosing was observed in Madagascar and taste was a barrier to use in Nepal.

It is noted that liquid chlorine was more often linked to long-term development approaches in endemic cholera contexts, including promotion (as compared to distribution), cost recovery, social marketing [22], local production [24], and vouchers [21].

### Combination Flocculant/Disinfectants

Combination flocculant/disinfectants, such as PUR Purifier of Water, are well suited to treat turbid water [25]. To use a PUR sachet, users add the sachet contents to 10 L of water, stir for 5 minutes, wait 5 minutes for the solids to settle, filter the water through a cloth into a second bucket, and wait 20 minutes before drinking.

PUR was evaluated in 3 contexts in 3 countries [17, 18, 26]: one low risk of bias [17], one medium risk of bias [26], and one high risk of bias [18]. In a randomized controlled trial (RCT) in an internally displaced persons camp in Liberia, 95% confirmed use was documented, along with a reduced diarrhea incidence of 67% (adjusted risk ratio [RR], 0.33; 95% confidence interval [CI], .30–.37) and reduced diarrhea prevalence of 77% (adjusted RR, 0.23; 95% CI, .21–.25) [26]. It is noted that households were provided all materials necessary to use PUR at no cost, received extensive training, and were visited weekly.

In a distribution with training in South Sudan, 78% of households could demonstrate all 5 steps to use PUR [18]. While >90% had confirmed use, PUR use could not be separated from Aquatab use, as both were distributed in the same hygiene kit. In Kenya, however, where PUR was distributed in an non-food item (NFI) kit with minimal training, only 2.3% of households could describe the 5 steps necessary for PUR, with a concurrent low reported use of 5.9% and confirmed use of 3.7% [17].

### Filtration

HWT filters include simple screens, ceramic, sand, and hollow-fiber filters; 2 studies were identified on filters [27, 28]. In a large study with a low risk of bias of >40000 people in an endemic cholera area in Bangladesh, 2 simple filters (a small nylon screen of 150 µm mesh size and a folded piece of sari cloth) were used in intervention groups and compared with a control group [27]. Cholera morbidity was reduced by approximately 40% in both the nylon and sari cloth filter groups (nylon filter RR, 0.59; sari cloth RR, 0.52), with >90% following the filtering instructions. After 5 years, participants were revisited, and in a study with a medium risk of bias, households in the sari cloth group were more likely to report use of some method of water treatment (35% compared with control at 23% and nylon group at 26%). Sari filter use was also identified to have a protective reduction in morbidity that extended to neighbors of filter users [28]. Filter use was identified as simple, improved water appearance and was culturally acceptable [27, 28].

### Solar Disinfection

Solar disinfection uses heat and ultraviolet radiation from the sun to inactivate bacteria, viruses, and protozoa in drinking water. Users place a clear container (ie, 1.5 L plastic bottle) on their roof in the sun for 6–48 hours, depending on amount of direct sunlight, and then drink the water.

SODIS was evaluated in one study with a high risk of bias in a development context in Kenya that led into an outbreak evaluation when cholera began in the project area [29]. The intervention consisted of the distribution of 1.5 L clear plastic bottles with instructions to give children <5 years old only SODIS-treated water. SODIS was effective at reducing self-reported diarrhea rates by 88% in children (odds ratio, 0.12; 95% CI, .02–.65; *P* = .014).

### Boiling

Promotion of boiling is not generally a common outbreak response strategy as it is energy intensive and does not provide residual protection of water during storage [6]. However, the materials for boiling are often available in the household, and previous education campaigns mean beneficiaries are often aware of boiling. In the evaluations described so far, boiling was not a promotional activity, but 14% of households reported boiling in DRC [16] compared with 81% in Madagascar [23]. Only one evaluation with a high risk of bias in Guinea-Bissau promoted boiling as a response intervention, as part of a hygiene campaign for cholera [31]. After the campaign, 40% of households reported boiling water; however, 66% reported using lemon to treat water (a local method that to our knowledge has not been evaluated). Additionally, no households reported consistent use of either method, and no confirmed use evidence was collected.

## DISCUSSION

The evaluations included in this review, in totality, present a moderate quality of evidence that HWT interventions can reduce the burden of disease in cholera outbreaks [26–29] and reduce the risk of disease transmission by improving the quality of household stored water [17, 18, 21, 23]. However, key program design and implementation characteristics are needed to ensure that HWT programs can reach this potential. These characteristics included appropriate training for the users of the product and community health worker (CHW) follow-up. Additionally, population-related barriers and facilitators affected uptake of HWT interventions in cholera outbreaks. These included the barrier of taste and odor resistance and the facilitators of prior exposure, ease of use, and links to preexisting development programming. These results have been incorporated into the Sphere Standards, which is a voluntary initiative to set minimum standards in humanitarian response with the aim of improving the quality of humanitarian assistance and the accountability of humanitarian actors [31]. The Sphere Standards now recommend only completing HWT programs if they are accompanied by appropriate training and follow-up.

In addition to the general results, results varied by HWT technology. Although the simplicity and ease-of-use of chlorine tablets were appreciated, low use was seen in NFI distributions with little training, having a storage container of appropriate size for the tablet was found to enable use, and having multiple tablets distributed in the same emergency was found to be confusing.

Liquid chlorine interventions included more long-term programs that use promotion, distribution, marketing, and voucher redemption. Previous exposure to liquid chlorine in development settings before an outbreak and links to development programming in the outbreak may have contributed to relatively higher use of liquid chlorine than chlorine tablets, which were predominantly distributed in NFI kits. It is noted that in one of the included studies, there was high use of chlorine tablets in a noncholera emergency evaluation (>90% confirmed use) where users had prior exposure to the tablets, the program existed before the emergency, and the tablets were distributed with CHW training and follow-up [17].

Overall, the most successful chlorine-based HWT programs in cholera outbreaks were effective in 3 areas: products, placement, and support [15]. Effective products have standardized dosage and instructions and are delivered with a safe storage container. Effective placement occurs where programs are directed at households familiar with the chlorination method before the emergency, implementing organizations have prior experience with the product, and thus there is high access to, demand for, and compliance with products. Effective support exists where implementing organizations provide the necessary supplies and training, and utilize community-based mobilization, education, and marketing techniques such as CHWs. The challenge, however, in chlorine-based HWT programs is balancing the competing criteria of (1) meeting the chlorine demand of the water; (2) maintaining FCR sufficient for disinfection during water distribution, transport, and household storage; and (3) not exceeding user taste and odor objections. In some cases, as seen by the taste and odor objections, this might not be possible.

For the products with fewer evaluations, with training PUR could be quite successful, although without training there was low retention of knowledge. Last, filtration, SODIS, and boiling HWT interventions were all implemented in nonacute endemic outbreak contexts. The quality of evaluation design is concurrently higher, but also difficult to generalize for other contexts.

Overall, HWT interventions were consistently reported to have potential to reduce the burden of cholera, if implemented appropriately. Of interest is that in the rerun search completed in April 2018, no peer-reviewed manuscript evaluating HWT interventions in a cholera outbreak was identified. The recent literature focuses on sustainability, integrating HWT into cholera prevention programs, and integration of HWT and oral cholera vaccine (OCV) programs.

A recent review found the sustainability of WASH interventions is frequently inadequately evaluated [32]. One study evaluated the sustainability of an existing development HWT with chlorine program that expanded after the Haiti earthquake and cholera outbreak [33]. Over 5 years, the program was monitored with 9832 supervisor and 80371 CHW visits. In 2010, 72.7% of supervisor visit records had positive FCR in household drinking water; this fell to 51.1% in 2014. These results documented a program with sustained, slightly decreasing household chlorination use over a period of 5 years, and inform discussions on the value of linking successful development programs to emergency relief, rehabilitation, and development.

The risk for cholera infection is >100 times higher for household contacts of cholera patients [34]. The CHoBI7 RCT in Bangladesh evaluated the impact of distribution of a hygiene kit including soap and chlorine tablets to households with a family member with cholera. Household contacts within the intervention group had 47% fewer cholera infections than controls, and intervention households had no stored drinking water with *V. cholerae* and 14 times higher odds of hand washing with soap at key events.

As OCV campaigns expand, there is interest in knowing how OCV campaigns impact WASH practices; results to date have been disparate. In Haiti, fewer postcampaign respondents reported treating and covering their drinking water and no significant changes in handwashing practice or cholera knowledge were reported 1 year after the campaign [35]. Conversely, in Papua New Guinea, respondents in vaccinated areas were more likely to have received cholera knowledge, and no significant differences in water, sanitation, and hygiene practices were observed 5 months after the campaign [36].

The main limitation of this work is that we could only include documents identified in the search strategy, and the gray literature search was not recompleted for the time period September 2016 to April 2018. It is possible there were additional gray literature studies that would meet inclusion criteria during this time. Two reasons are postulated for this lack of recent data: (1) The evidence is sufficient to guide programming [31]; and (2) the largest current cholera outbreak is in Yemen, where access is restricted and it is difficult to conduct monitoring and evaluation of programs [3].

Further research is recommended to determine local barriers and facilitators to HWT uptake for specific cholera contexts; the impact of HWT filters on cholera; how to scale up existing development HWT programs in the advent of cholera; the sustainability of HWT programs implemented in cholera outbreaks; the impact of integrating HWT and OCV campaigns; and how to complete monitoring of HWT programs in low-access conflict emergencies.

## Supplementary Data

Supplementary materials are available at *The Journal of Infectious Diseases* online (http://jid.oxfordjournals.org/). Supplementary materials consist of data provided by the author that are published to benefit the reader. The posted materials are not copyedited. The contents of all supplementary data are the sole responsibility of the authors. Questions or messages regarding errors should be addressed to the author.

Supplementary Table

## Notes

***Financial support.*** The larger systematic review from which this data on HWT was gleaned was supported by the Humanitarian Evidence Programme, and a portion of the search was supported by 3ie, the International Initiative for Impact Evaluation.

***Supplement sponsorship.*** This work is part of a supplement sponsored by the Bill and Melinda Gates Foundation.

***Potential conflicts of interest.*** D. L. has received grant support at Tufts to evaluate novel household water filters and purifiers in low-income, nonemergency contexts. Both authors have conducted research on HWT options in cholera outbreaks, and note their research was subject to the same search criteria as all identified research. All authors have submitted the ICMJE Form for Disclosure of Potential Conflicts of Interest. Conflicts that the editors consider relevant to the content of the manuscript have been disclosed.

Presented in part: Emergency Environmental Health Forum, Kathmandu, Nepal, November, 2016; Water and Health: Where Science Meets Policy, UNC Chapel Hill, NC, October, 2017; and published in the practitioner journal *Waterlines* 37:5.
