# Supplementary material for: Household Water Treatment and Cholera Control
Source: J Infect Dis. 2018 Sep 11;218(Suppl 3):S147–53. doi: 10.1093/infdis/jiy488 (PMC6188534; doi:10.1093/infdis/jiy488)
Supplement: Supplementary Table [file jiy488_suppl_supplementary_table.docx]

TABLE S1. Detailed information on each included study

| **Author and Title** | **Context** | **Description of Activities** | **Evaluation** | **Key Impacts** | **Bias** | |  |
| --- | --- | --- | --- | --- | --- | --- | --- |
| ACF (2009) Household NFI monitoring Report (PDM) May 2009  *Grey Literature* | Cholera – Outbreak  Zimbabwe | **CHLORINE TABLETS**  Aquatabs® distributed to HH as part of an NFI kit with bucket and lid (~33,000 – kits, other contents not described) | Quantitative  Cross-sectional: 218 HH (Random) | 26% of HH reported use  17% of HH confirmed use (> 0.5 mg/L)  Low Aquatab® use because water was collected from a borehole 'safe water’  75% of HH used the bucket  Overdosing, with smell and taste being issues. | High Risk of Bias  Inconsistent reporting, self-reported information, FCR was measured but not fully reported. | |  |
| ACF (2014) Hygiene Kits Post Distribution Monitoring Report  *Grey Literature* | Cholera – Outbreak  South Sudan | **CHLORINE TABLETS**  Aquatabs distributed in NFI kits to 7,348 HH. Kit also included: bucket, PuR® Purifier of Water packets and filter cloth | Quantitative  Cluster Cross-sectional: 351 HH | 87% confirmed use (>0.1 mg/L) in HH with Aquatabs (6% of HH FCR >0.5 mg/L)  >90% of HH had FCR in Juba (range 83-100%)  78% of HH could demonstrate correct use of PuR  HH without FCR said they get water from a treated tanker, or are saving the Aquatabs for when cholera outbreaks again. | High Risk of Bias  Inconsistent reporting. Spillover effects likely. | |  |
| ACF - Tokplo (2015)  Projet de reprise communautaire de la lutte contre le choléra et les maladies hydriques dans les zones de santé de Minova (Sud Kivu) et de Kirotshe (Nord Kivu), R.D. Congo  *Grey Literature* | Cholera – Endemic  D.R. Congo | **CHLORINE TABLETS**  Chloramine tablets with hygiene promotion | Quantitative  Before/After: 384 HH | 14% reported use of tablets.  14% confirmed use (54/ 54 HH had FCR 0.3-0.6 mg/L)  Reduction from 11 to 0 and from 30 to 7 cholera cases (monthly basis) in the intervention areas | Low Risk of Bias  Well-defined sampling strategy; limitations clearly stated | |  |
| Lantagne (2012) Use of Household Water Treatment and Safe Storage Methods in Acute Emergency Response: Case Study Nepal  *Published* | Cholera – Outbreak  Nepal | **CHLORINE TABLETS**  Local NGOs using pre-positioned stock. 1565 HH – received Aquatabs® but also liquid chlorine (Water Guard, Piyush) | Quantitative  Cross-sectional: 400 HH | 8.3% reported use (Liquid Chlorine: WaterGuard: 6.3% Piyush: 15.8%)  6.8% confirmed use (FCR ≥0.2 mg/L) (Liquid Chlorine: WaterGuard: 3.5%; Piyush: 8.3%) | Low Risk of Bias  Spillover between several similar interventions | |  |
| Lantagne (2012) Use of Household Water Treatment and Safe Storage Methods in Acute Emergency Response: Case Study Kenya  *Published* | Cholera – Outbreak  Kenya | **CHLORINE TABLETS**  Pre-positioned stock. Distribution of Aquatabs® and PuR® Purifier of Water in an NFI kit to 5,592 HH. | Quantitative  Cross-sectional: 409 HH | 12.7% reported use (PuR® Purifier of Water: 5.9%)  7.9% confirmed use (PuR®: 3.7%) (FCR ≥0.2 mg/L)  5.3% effective use <1 CFU/100mL (PuR: 2.3%) | Low Risk of Bias  Selection bias not likely, consistent reporting of outcomes | |  |
| Unicef - Ruiz-Roman (2009) Evaluation of the blanket distribution of non-food items as part of the cholera response in Zimbabwe  *Grey Literature* | Cholera  Zimbabwe  Outbreak | **CHLORINE TABLETS**  ~200,000 HH NFI distribution (1 - 20L bucket, 1 - 20L bucket w tap, 30 - water purification tablets, 3 ORS sachets and 1 pack of IEC materials) | Quantitative  Evaluation: 307 HH | 87% of 307 surveyed HH reported receiving a hygiene kit; only 33% reported receiving all 5 recommended items (differences in kits)  59% of HH requested additional quantities – mostly from families of 6 or more  Soap was most used item | High Risk of Bias  Spillover effects likely, selective reporting | | |
| ACF (2014) Projet pilote de l'approche de marché pour la promotion du chlore liquide  *Grey Literature* | Cholera – Endemic  D.R. Congo | **CHLORINE LIQUID**  Promotion and distribution of liquid chlorine with vouchers to 834 HH. | Quantitative  Cross-sectional: 32 HH | *No reported use*. Voucher redeemed by 88% of HH  69% confirmed use (FCR ≥0.2 mg/L; Average FCR 0.5 mg/L)  97% of HH (31/32) reported being satisfied with liquid chlorine as a HWT | | Medium Risk of Bias  Potential spillover and selective reporting | |
| Dunston (2001) Collaboration, cholera, and cyclones: A project to improve point-of-use water quality in Madagascar  *Published* | Cholera – Outbreak  Madagascar | **CHLORINE LIQUID**  Liquid Chlorine marketed to community (Safe Water System-WaterGuard). Jerry cans available but not distributed. | Quantitative  Before/After: 375 HH – 15 communities stratified by mobilization strategy | 19.7% reported use (increased from 8.4% baseline, 6 months after mobilization dropped to 11.2%)  *No confirmed use* - FCR in HH using SwS 0.23 mg/L (median), compared to 0.1 mg/L in HH not using (p=0.005) | | High Risk of Bias  Selective reporting, incomplete outcomes. | |
| Lantagne (2012) Use of Household Water Treatment and Safe Storage Methods in Acute Emergency Response: Case Study Nepal  *Published* | Cholera – Outbreak  Nepal | **CHLORINE LIQUID**  Local NGOs using pre-positioned stock. 1565 HH – received liquid chlorine (WaterGuard®, Piyush®) but also Aquatabs®. | Quantitative  Cross-sectional: 400 HH | 22.2% reported use (2 products: WaterGuard®: 6.3% Piyush®: 15.8%) (Aquatabs®: 8.3%)  11.8% confirmed Use (2 products: WaterGuard®: 3.5%; Piyush®: 8.3%) (Aquatabs®: 6.8%) (FCR ≥0.2 mg/L) | | Low Risk of Bias  Selection bias not likely, clear and consistent reporting of outcomes | |
| Mong (2001)  Impact of Safe Water System on Water Quality in Cyclone-Affected Communities in Madagascar  *Published* | Cholera – Outbreak  Madagascar | **CHLORINE LIQUID**  Liquid chlorine and 5 gallon flexible jerry can distributed to 11,700 HH with some education about use. | Quantitative  123 HH (random) | 65% reported use (n=123); ‘ever used’ 85%; SwS already promoted in the area  45% confirmed use (n=40) (FCR ≥0.2 mg/L)  76% report receiving jerry can; 76% reported using | | High Risk of Bias  Selective reporting and outcomes. | |
| Date (2013) Evaluation of a Rapid Cholera Response Activity—Nyanza Province, Kenya, 2008  *Published* | Cholera  Kenya  Endemic | **CHLORINE LIQUID**  Distribution of HWT and hygiene kits (not described); environmental investigations, cholera case management. | Quantitative  Cross-sectional: 358 intervention HH and 365 control HH | Social contacts (friends, family, and neighbours), which suggests that social networks can be a valuable resource.  *No reported use* (Reported any water treatment: Intervention: Control 56%: 37%; p<0.001)  *No confirmed use* (‘Detectable’ FCR 17% in intervention and 14% in control groups; NS) | | High Risk of Bias  Intervention overlap, intervention loosely described, convenience sample, 3 month recall time | |
| ACF (2014) Hygiene Kits Post Distribution Monitoring Report  *Grey Literature* | Cholera – Outbreak  South Sudan | **PUR**  Aquatabs® distributed in NFI kits to 7,348 HH. Kit also included: bucket, PuR® Purifier of Water packets and filter cloth. | Quantitative  Cluster Cross-sectional: 351 HH | >90% of HH had FCR in Juba (range 83-100%) (PuR or Aquatabs)  78% of HH could demonstrate correct use of PuR  HH without FCR said they get water from a treated tanker, or are saving the Aquatabs for when cholera outbreaks again. | | High Risk of Bias  Inconsistent reporting. Spillover effects likely. | |
| Doocy (2006) Point-of-use water treatment and diarrhoea reduction in the emergency context: an effectiveness trial in Liberia  *Published* | Cholera  Liberia  Endemic | **PUR**  PuR® Purifier of Water sachets (weekly distributions) with 2 10 L buckets compared to HH given just buckets. | Quantitative  200 HH intervention and 200 HH control | 95.4% confirmed use – “compliant” with FCR and reported use  Diarrhoea incidence reduced by 67% (ARR 0.33; 95%CI 0.30-0.37); diarrhoea prevalence reduced by 77% (ARR 0.23; 95%CI 0.21-0.25). Covered stored water alone was also protective for diarrhoea incidence (ARR 0.84; 95%CI 0.82-0.86).  Improved visual appearance and taste from PuR group | | Medium Risk of Bias  Weekly visits for 12 weeks prone to courtesy bias; rainy season over – less diarrhoea. | |
| Lantagne (2012) Use of Household Water Treatment and Safe Storage Methods in Acute Emergency Response: Case Study Kenya  *Published* | Cholera - Outbreak  Kenya | **PUR**  Pre-positioned stock. Distribution of Aquatabs® and PuR® Purifier of Water in an NFI kit to 5,592 HH. | Quantitative  Cross-sectional: 409 HH | 5.9% reported use  3.7% confirmed use (FCR ≥0.2 mg/L)  2.3% effective use <1 CFU/100mL | | Low Risk of Bias  Selection bias not likely. Clear and consistent reporting of outcomes. | |
| Colwell (2003) Reduction of cholera in Bangladeshi villages by simple filtration  *Published* | Cholera  Bangladesh  Endemic | **FILTRATION**  Simple filter intervention group compared to control. Intervention groups: 1) Nylon mesh water filter 150- µm mesh size and 2) folded sari cloth as a filter. | Quantitative  65 villages: 27 Villages using Sari; 25 Villages using filter screen; 13 villages control. ~44,000 in each group. | 90% reported use of filters  Health impact: 38% reduction in cholera cases by filter use, hospital confirmed cases. (Nylon filter: control OR: 0.59; p<0.05) (Cloth filter: control OR: 0.52 Sari (8 folds); p<0.05) | | Low Risk of Bias  Pilot intervention had strong consistent results, but increased for power | |
| Huq (2010) Simple sari cloth filtration of water is sustainable and continues to protect villagers from cholera in Matlab, Bangladesh  *Published* | Cholera  Bangladesh  Endemic | **FILTRATION**  5 years after Colwell, revisit same HH to see use of HWT | Quantitative  7,233 HH, 5 years after Colwell (2003); 2,251 nylon filter, 2,556 cloth group, and 2,426 control group intervention. | 31% reported use of a filter (2207 of 7233 HH); Sari group (35%), Nylon filter (26%), control group (23%)  Confirmed use 38% of reported rates (19/50) (through 11 hour observation period) | | Medium Risk of Bias  Spillover effects likely | |
| Conroy (2001)  Solar disinfection of drinking water protects against cholera in children under 6 years of age  *Published* | Cholera - Outbreak  Kenya | **SODIS**  1.5L clear plastic bottle distributed with instructions (SODIS project) – targeted children under <5 | Quantitative  67 HH intervention and 64 control; HH had child under 5 years for original study then monitored a year after (Case-control out of an RCT) | *No reported use.* (67/131 used SODIS)  Health impact: Self-reported cases of cholera: <6 yr: (RR 0.12; 0.02-0.65; p=0.014); 6-15 yr: (RR 1.09; 0.58-2.05); Adults: (RR 1.2; 0.59-2.5) | | High Risk of Bias  Inconsistent results, unclear intervention impact | |
| Einarsdbttir (2001) Health Education and Cholera in Rural Guinea-Bissau  *Published* | Cholera  Guinea-Bissau  Endemic | **BOILING**  Hygiene promotion to support treating water (and other hygiene practices). Radio, TV, health staff, poster, word-of-mouth, song, theatre group | Quantitative  53 HH (Random) | 66% reported use with lemon to treat water; 40% reported boiling water; no one reported only drinking treated (boiled /lemon) water. Not consistent use of treated water. | | High Risk of Bias  Small sample size, open-ended questions, self-reported results | |
